# The impact of central venous catheter use on long-term growth and neurobehavioral outcome in preterm children born < 29 weeks of gestation

**DOI:** 10.1186/s40348-026-00247-y

**Published:** 2026-06-23

**Authors:** Christine Silwedel, Jana Retzmann, Ingmar Fortmann, Anne Grimm, Anna Häfke, Fabian Kleindiek, Kathrin Hanke, Cornelia Wiechers, Ursula Felderhoff-Müser, Harald Ehrhardt, Jochen Essers, Egbert Herting, Juliane Spiegler, Wolfgang Göpel, Christoph Härtel

**Affiliations:** 1 Department of Pediatrics, University Hospital Würzburg, University of Würzburg, Josef-Schneider-Str. 2, 97080 Würzburg, Germany; 2 Department of Pediatrics, University of Lübeck, Lübeck, Germany; 3 University Hospital of Schleswig-Holstein, Campus Lübeck, Lübeck, Germany; 4 Department of Neonatology, Tübingen University Children’s Hospital, Tübingen, Germany; 5 Department of Paediatrics I, University Hospital Essen, University of Duisburg-Essen, Essen, Germany; 6 C-TNBS Center for Translational Neuro- and Behavioral Sciences, Essen, Germany; 7 Department of Neonatology, Charité – Universitätsmedizin Berlin, Berlin, Germany; 8 German Centre for Child and Adolescent Health (DZKJ), partner site Berlin, Berlin, Germany; 9 Division of Neonatology and Pediatric Intensive Care Medicine, Department of Pediatrics and Adolescent Medicine, University Medical Center Ulm, Ulm, Germany

**Keywords:** Preterm infant, Central venous catheter, German neonatal network, Weight gain, Neurodevelopment, Early enteral nutrition

## Abstract

**Background:**

Preterm infants often receive central venous catheters (CVCs) for parenteral nutrition during initial enteral feeding advancement. Whether the use of CVCs promotes growth and neurodevelopmental outcomes is currently unknown.

**Methods:**

Using German Neonatal Network data, this retrospective population-based cohort study evaluated long-term outcomes in very low birth weight infants (VLBWI) born < 29 weeks of gestation. Multivariate regression analyses and propensity score matching were applied to assess associations between CVC use, somatic growth, and cognitive outcomes (Wechsler Preschool and Primary Scale III intelligence quotient (IQ), Strengths and Difficulties Questionnaire (SDQ)) at 5–7 years of age.

**Results:**

In the follow-up cohort of 2072 infants, 74% had a history of neonatal CVC use. VLBWI with CVCs had a significantly lower mean gestational age (25.97 vs. 26.97 weeks, *p* < 0.001) and lower somatic parameters at birth (mean weight: 829 vs. 986 g, length: 33.7 vs. 35.7 cm, head circumference: 23.8 vs. 25.1 cm, all: *p* < 0.001) than those without CVCs. Before risk adjustment, mean weight, head circumference, and body mass index at preschool age were significantly lower in children with prior CVC use. These children additionally exhibited lower IQ and higher SDQ scores. Adjusting for potential confounders, linear regression analyses and propensity score matching indicated that CVC use itself was not associated with differences in weight, length, head circumference, or cognitive outcomes at preschool age. Notably, In infants with CVCs, full enteral feeding was achieved approximately 8 days later than in those without CVCs (21.5 vs. 13.2 d, *p* < 0.001). Prolonged attainment of full enteral feeding was associated with reduced preschool IQs (β: -0.069, CI: -0.116 to -0.022, *p* < 0.001).

**Conclusion:**

In this study, CVC use was not associated with improved somatic growth or psychomotor outcomes at preschool age. Furthermore, our data suggest that earlier attainment of full enteral feeding is associated with more favorable long-term psychomotor outcomes. Within the limits of an association study, these findings support prioritizing strategies that facilitate early enteral feeding advancement in VLBWI, while avoiding routine CVC insertion whenever clinically appropriate.

**Supplementary Information:**

The online version contains supplementary material available at 10.1186/s40348-026-00247-y.

## Background

Advances in perinatal medicine have markedly improved survival among very low birth weight infants (VLBWI, infants with a birth weight < 1,500 g). However, the risk of long-term morbidities – including neurodevelopmental impairment – remains substantial [1, 2]. Optimal nutritional support in early life has emerged as a key modifiable factor with potential impact on growth, morbidity, and long-term development. Previous studies suggest that early provision of energy and nutrients may promote growth and improve long-term neurodevelopmental trajectories [3, 4]. Accordingly, guidelines such as those from the European Society for Paediatric Gastroenterology, Hepatology and Nutrition (ESPGHAN) endorse proactive nutritional strategies in VLBWI [5, 6].

In clinical practice, VLBWI commonly receive parenteral nutrition accompanying a gradual advancement of enteral feeding. Vascular access is provided either via peripheral venous catheters or by insertion of central venous catheters (CVCs). CVCs are often used to facilitate high parenteral intakes of amino acids, lipids and other essential nutrients within the target daily fluid volume, to provide reliable vascular access, and to limit the number of venipuncture procedures [7]. However, their use has also been associated with an increased risk of blood stream infection (BSI) and other serious complications [8, 9].

Given the central role of early-life stressors and inflammatory insults in shaping long-term neurodevelopment, concerns persist that the complications associated with CVC use may counteract the intended benefits of optimized parenteral nutrition and minimized handling through secure vascular access. Furthermore, evidence evaluating the impact of highly dosed parenteral nutrition requiring a CVC on long-term growth and neurodevelopment remains sparse.

## Methods

This study aimed to investigate the relationship between CVC use during the first hospital stay and long-term outcome, including growth and cognition as well as behavioral performance in preterm born children.

### Study population

The German Neonatal Network (GNN) is a comprehensive, population-based observational multicenter cohort study enrolling VLBWI across 71 tertiary level neonatal intensive care units in Germany, with the coordinating center based in Lübeck. The study aims to investigate risk factors associated with preterm birth and preterm morbidity as well as long-term outcome of preterm neonates. It adheres to all ethical standards, having received approval from the University of Lübeck’s ethics committee (#08–022) and local committees at all participating sites. Enrollment requires written informed parental consent, and the lack thereof as well as lethal malformations are exclusion criteria. Clinical data are systematically collected using case record forms, coded, stored in a central database, and evaluated at the coordinating center. High-quality data collection is ensured by annual on-site monitoring.

This study, conducted within the GNN framework, focuses on a cohort of VLBWI born between 22 + 0 and 28 + 6 weeks of gestation, enrolled between 2009 and 2016. A subgroup of these infants was invited to participate in a comprehensive and standardized follow-up assessment at 5–7 years of age and formed the study population for the present analysis. Follow-up evaluations were performed by the same study team across the participating sites, thus limiting inter-observer variability. The assessment included detailed tests of organ function, growth, neurodevelopment, and behavior. Infants were categorized according to whether they had received a CVC during their initial hospitalization, or if they had been managed using exclusively peripheral venous access.

### Primary outcome

The primary outcome was cognitive abilities at preschool age, assessed during a standardized follow-up at preschool age using the Wechsler Preschool and Primary Scale – third edition (WPPSI-III). The WPPSI-III evaluates verbal and performance domains and provides a composite full-scale intelligence quotient (IQ) score, with higher values corresponding to greater intellectual capacity.

### Secondary outcomes

Secondary outcomes included social behavior as well as somatic development at preschool age. The Strengths and Difficulties Questionnaire (SDQ) [10] was used to evaluate the parental perception of their children’s behavior. This tool comprises 25 items across 5 subscales (1. emotionality, 2. conduct, 3. hyperactivity / inattention, 4. peer relationships, 5. prosocial behavior). Responses are rated on a 3-point Likert scale. The total SDQ score is calculated using 20 questions across subscales 1–4 and ranges between 0 and 40, with higher scores indicating more significant behavioral concerns. A total score of ≥ 16 indicates mental health difficulties [11]. Somatic parameters assessed at preschool age included weight, length, and head circumference.

### Definitions

The gestational age in weeks + days was determined from prenatal obstetric ultrasound and examination. Weight (g / kg), length (cm), and head circumference (cm) were measured at birth and during follow-up at 5–7 years of age. Body mass index (BMI) at follow-up (kg/m^2^) was calculated by dividing weight (kg) by height squared (m^2^). BMI z-scores were obtained by comparing individual BMI values with age- and sex-specific reference percentiles published previously [12] and adjusting for gestational age using the LMS method [13]. Small for gestational age (SGA) was defined as a birth weight below the 10th percentile for the corresponding gestational age [14]. CVC was defined as any indwelling catheter placed in a central vein, including umbilical venous and peripherally inserted central catheters. Full enteral feeding was defined as the first day on which an enteral intake of ≥ 150 ml/kg body weight was tolerated. Clinical sepsis was determined according to the criteria of the German national nosocomial infection surveillance system in preterm infants (NEO-KISS), based on the presence of clinical and laboratory indicators of infection and antibiotic therapy ≥ 5 days [15]. Accordingly, culture-proven sepsis was characterized by meeting the clinical sepsis criteria along with the identification of a causative pathogen in one or more blood cultures [15]. Necrotizing enterocolitis (NEC) was defined as necrotizing inflammation of the intestinal tract, diagnosed according to clinical and radiological signs (Bell > stage 2a) or surgical and histological confirmation. Focal (spontaneous) intestinal perforation (FIP) was determined based on clinical, surgical, and histological findings. Intraventricular hemorrhage (IVH) was diagnosed and classified using cranial ultrasound [16].

Infants requiring surgical intervention for NEC or FIP were excluded from this study, as these conditions are confounding variables concerning feeding modes and primary outcome.

### Statistical analysis

Statistical analysis was conducted using IBM SPSS Statistics (version 29.0.2; IBM Corp., Armonk, NY). Missing data were not imputed. Baseline characteristics were described using means with standard deviation (SD) for continuous variables and the number (n) of children and percentage (%) for nominal variables. Univariate analyses were performed using the Chi-square test or the independent t-test as appropriate, with a significance threshold set at *p* < 0.05 for individual tests.

Multiple linear regression models were used to evaluate the association between CVC use and continuous long-term outcome measures. In model 1, covariates included gestational age, birth weight, SGA status, clinical or culture-confirmed sepsis, CVC use, and IVH. For analysis of neurodevelopmental long-term outcomes, parental educational levels were additionally included as independent variables (model 2). In separate regression analyses assessing the association between feeding advancement and outcomes, age at full enteral feeding was additionally incorporated into both models 1 and 2, resulting in models 4 and 5, respectively.

To further address interrelated covariates and reduce the influence of unmeasured confounding, we conducted subsequent analyses in propensity score matched infants (1:1 matching) using the following matching parameters: sex, gestational age, SGA status, maternal educational level, and IVH (model 3). Matching quality was evaluated by assessing standardized mean differences (SMDs) for all covariates before and after matching, with SMDs < 0.1 considered indicative of adequate covariate balance between groups [17].

## Results

### Study cohort

**Fig. 1:**
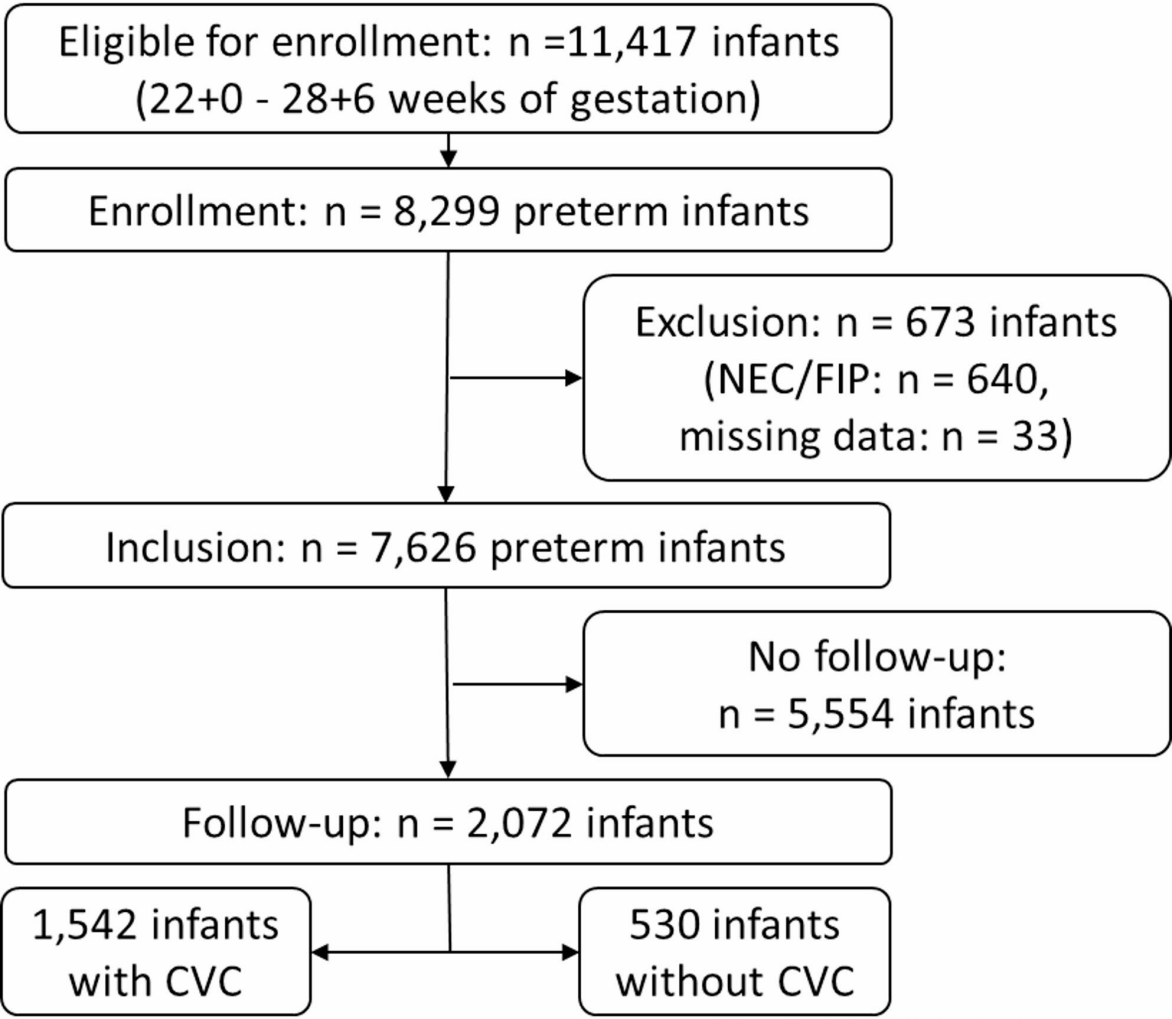
Flow diagram of infants enrolled in this study

After exclusion of 673 infants due to intervention for NEC / FIP or missing data, 7626 infants born at 22 + 0–28 + 6 weeks of gestation were included in the primary cohort. *N* = 2072 of these infants received a follow-up assessment at preschool age and were included in this study (Fig. 1).

In the study cohort, *n* = 1080 (52.1%) infants were male and *n* = 992 (47.9%) were female, with an average gestational age of 26.22 (SD: ±1.51) weeks and a mean birth weight of 869.5 (± 238.81) g (Table 1).

**Table 1:** Clinical characteristics of infants included in the study

Outcome		Total (*n* = 2072; 100%)	Without CVC (*n* = 530; 25.4%)	With CVC (*n* = 1542; 74.4%)	P*
Perinatal and neonatal period	Gestational age, mean (SD), weeks	26.22 (± 1.51)	26.97 (± 1.12)	25.97 (± 1.55)	**< 0.001**
	Birth weight, mean (SD), g	869.53 (± 238.81)	986.44 (± 228.55)	829.29 (± 228.81)	**< 0.001**
	Length at birth, mean (SD), cm	34.23 (± 3.33)	35.70 (± 3.04)	33.73 (± 3.27)	**< 0.001**
	Head circumference at birth, mean (SD), cm	24.11 (± 2.13)	25.08 (± 1.89)	23.78 (± 2.11)	**< 0.001**
	SGA status, n (%)	266 (12.8)	38 (7.2)	228 (14.8)	**< 0.001**
	Sex				0.182
	Female, n (%)	992 (47.9)	267 (50.4)	725 (47.0)	
	Male, n (%)	1080 (52.1)	263 (49.6)	817 (53.0)	
	Multiple birth, n (%)	737 (35.6)	190 (35.8)	547 (35.5)	0.876
	Sepsis (clinical), n (%)	826 (39.9)	156 (29.4)	670 (43.5)	**< 0.001**
	Sepsis (culture proven), n (%)	334 (16.1)	57 (10.8)	277 (18.0)	**< 0.001**
	IVH, n (%)	467 (22.5)	75 (14.2)	392 (25.4)	**< 0.001**
	Begin enteral feeding, mean (SD), d	1.20 (± 0.59)	1.08 (± 0.33)	1.23 (± 0.66)	**< 0.001**
	Full enteral feeding, mean (SD), d	19.37 (± 15.02)	13.18 (± 8.02)	21.52 (± 16.25)	**< 0.001**
	Venous access, mean (SD), d	24.74 (± 18.12)	15.28 (± 9.26)	27.64 (± 19.15)	**< 0.001**
	Length of hospital stay, mean (SD), d	87.37 (± 31.30)	73.41(± 21.94)	92.21 (± 32.61)	**< 0.001**
Follow-up	Weight at follow-up, mean (SD), kg	18.76 (± 3.54)	19.19 (± 3.41)	18.61 (± 3.57)	**< 0.001**
	Body length at follow-up, mean (SD), cm	112.81 (± 6.14)	113.18 (± 6.04)	112.69 (± 6.18)	0.113
	Head circumference at follow-up, mean (SD), cm	50.15 (± 1.96)	50.65 (± 1.81)	49.98 (± 1.97)	**< 0.001**
	BMI at follow-up, mean (SD), kg/m^2^	14.64 (± 1.68)	14.88 (± 1.59)	14.55 (± 1.70)	**< 0.001**
	Z-score BMI at follow-up, mean (SD)	0.01 (± 1.08)	0.21 (± 0.99)	-0.05 (± 1.10)	**< 0.001**
	IQ at follow-up, mean (SD)	97.27 (± 13.26)	99.78 (± 12.49)	96.35 (± 13.31)	**< 0.001**
	SDQ at follow-up, mean (SD)	9.95 (± 5.54)	9.14 (± 5.13)	10.22 (± 5.63)	**< 0.001**

Bold values indicate statistically significant differences (*p* < 0.05)

*SD *standard deviation, *SGA *small for gestational age, *IVH *intraventricular hemorrhage, *BMI *body mass index (z-scores adjusted for gestational age), *IQ *intelligence quotient, *SDQ *strengths and difficulties questionnaire

* Chi-square-test or independent t-test, significance between infants with and without CVC. % is given for the respective cohort (total / without / with CVC)

### CVC use in the neonatal period is determined by perinatal risk profile

In the follow-up cohort, 74% of all infants had a history of CVC use during their primary hospital stay. When assessing neonatal characteristics, significant differences were observed between the groups with and without CVCs. Infants with CVCs were characterized by lower gestational age (mean: 25.97 vs. 26.97 weeks, *p* < 0.001) and birth weight (829.3 vs. 986.4 g, *p* < 0.001), as well as reduced body length (33.7 vs. 35.7 cm, *p* < 0.001) and head circumference (23.8 vs. 25.1 cm, *p* < 0.001) at birth compared to those without CVCs (Table 1). Furthermore, a greater proportion of infants in the CVC group were classified as SGA (14.8 vs. 7.2%, *p* < 0.001) (Table 1). Clinical and culture proven sepsis occurred more frequently in the CVC cohort, with rates of 43.5 vs. 29.4% and 18 vs. 10.8%, respectively (*p* < 0.001). Similarly, IVH was more common among infants with CVCs (25.4 vs. 14.2%, *p* < 0.001) (Table 1). Significant differences between infants with and without CVCs were furthermore observed in the duration of intravenous access (27.6 vs. 15.3 d, *p* < 0.001) and the length of hospital stay (92.2 vs. 73.4 d, *p* < 0.001) (Table 1).

### CVC use is not associated with improved growth and neurobehavioral outcome

Standardized follow-up assessment showed that preterm children with a history of CVC use had a significantly lower body weight (mean: 18.6 vs. 19.2 kg, *p* < 0.001) and head circumference (50.1 vs. 50.7 cm, *p* < 0.001) at preschool age, as well as lower gestational age-adjusted BMI z-scores (-0.05 vs. 0.21, *p* < 0.001) compared to the cohort without prior central venous access (Table 1). They also exhibited lower IQ scores (97.3 vs. 99.8) and higher SDQ scores (10.2 vs. 9.1) at preschool age. Using a multivariable regression approach with adjustment for risk factors associated with deficits in growth or neurological development, we could not confirm a significant association between CVC use and long-term weight, length, head circumference, IQ and SDQ outcomes in preterm children (Table 2). A history of CVC use was linked with a modest decrease in age-adjusted BMI z-scores (β = -0.123, 95% confidence interval (CI): -0.233 to -0.012, *p* = 0.030) (Table 2).

**Table 2:** Multiple linear regression models showing association of outcomes with CVC use after adjusting for potential confounding variables

	Outcome	Model 1		Model 2	
		β (95% CI)	p	β (95% CI)	p
Preschool age	Weight (kg)	0.018 (-0.331 to 0.368)	0.917		
	Length (cm)	0.554 (-0.052 to 1.159)	0.073		
	Head circumference (cm)	-0.090 (-0.268 to 0.088)	0.322		
	BMI (kg/m^2^)	-0.122 (-0.293 to 0.049)	0.163		
	Z-score BMI	-0.123 (-0.233 to -0.012)	**0.030**		
	IQ			-0.136 (-1.607 to 1.334)	0.856
	SDQ			-0.068 (-0.709 to 0.573)	0.836

Linear regression analysis was conducted to evaluate the association between central venous catheter (CVC) use and weight, length, head circumference, body mass index (BMI) and BMI z-scores adjusted for gestational age, as well as intelligence quotient (IQ) and strengths and difficulties questionnaire (SDQ) scores at preschool age. Independent variables included in model 1: gestational age, birth weight, small for gestational age, CVC, sepsis, and intraventricular hemorrhage. Model 2 included the independent variables listed for model 1 plus parental educational level. Results are presented as β coefficients with 95% confidence intervals (CI) and corresponding p-values. Bold values indicate statistically significant differences (*p* < 0.05). N = 2049 for weight, n = 2062 for length, n = 2065 for head circumference, n = 2041 for BMI, n = 1996 for z-score BMI, n = 1489 for IQ, and n = 1539 for SDQ. Full regression analyses are provided in additional Tables 1, 2 and 3

To account for interrelated variables and potential hidden confounders, we additionally conducted propensity score matching in pairs of infants with and without CVCs (Additional Table 4). After propensity score matching, covariate balance was substantially improved, with SMDs ranging from − 0.02 to 0.04 across all models (Additional Table 4). Between 412 and 488 infant pairs were retained in the matched analyses, depending on the model. In the matched cohort, children without neonatal CVC utilization had a significantly higher BMI and, accordingly, higher BMI z-scores in comparison to those with a history of CVC use (BMI: 14.9 vs. 14.7 kg/m^2^, *p* = 0.019, z-scores BMI: 0.20 vs. 0.02, *p* = 0.003). The effect size, however, was small (Cohen’s d 0.11 and 0.14, respectively) (Table 3).

**Table 3:** Association of outcomes with CVC use in a cohort of propensity score matched infants (model 3)

	Outcome	Mean ± SD (without CVC)	Mean ± SD (with CVC)	Mean diff	95% CI	P*	Cohen’s d
Preschool age	Weight (kg)	19.2 ± 3.4	18.8 ± 3.3	0.371	0.000 to 0.741	0.050	0.089
	Length (cm)	113.1 ± 6.1	113.0 ± 5.9	0.120	-0.568 to 0.807	0.732	0.016
	Head circum-ference (cm)	50.6 ± 1.8	50.4 ± 1.8	0.176	-0.021 to 0.374	0.080	0.080
	BMI (kg/m^2^)	14.9 ± 1.6	14.7 ± 1.6	0.213	0.034 to 0.391	**0.019**	0.107
	Z-score BMI	0.20 ± 1.00	0.02 ± 1.00	0.178	0.062 to 0.294	**0.003**	0.139
	IQ	99.7 ± 12.5	99.4 ± 12.5	0.307	-1.281 to 1.895	0.704	0.018
	SDQ	9.2 ± 5.2	9.2 ± 5.3	-0.002	-0.733 to 0.728	0.995	0.000

Parameters used for propensity score matching included sex, gestational age, small for gestational age, maternal educational level, and intraventricular hemorrhage. Full matching was confirmed using standardized mean differences (Additional Table 4)

*SD *standard deviation, *diff* difference, *CI *confidence interval, *BMI* body mass index (z-scores adjusted for gestational age), *IQ *intelligence quotient, *SDQ *strengths and difficulties questionnaire

*independent t-test, significance between matched infants with and without CVC

Bold values indicate statistically significant differences (*p* < 0.05)

### Later attainment of full enteral feeding is associated with reduced IQ

In infants with CVCs, full enteral feeding was achieved approximately 8 days later than in those without CVCs (21.5 vs. 13.2 d, *p* < 0.001), and regression analyses confirmed that CVC use was independently associated with a longer time to full enteral feeding (Table 1 and Additional Table 5). Using multiple regression models, we evaluated the association between enteral feeding advancement and long-term outcome. Our analysis revealed that a longer time to attainment of full enteral feeds correlated with a lower preschool IQ (β: -0.069, 95% CI: -0.116 to -0.022, *p* < 0.001) (Table 4), corresponding to an estimated decrease of approximately 0.07 IQ points per additional day until full enteral feeding.

**Table 4:**
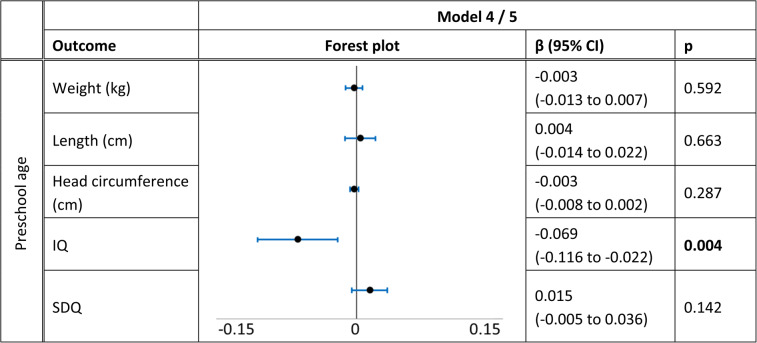
Multiple linear regression analysis showing association of long-term outcomes with enteral feeding advancement

Linear regression analysis was conducted to evaluate weight, length and head circumference as well as intelligence quotient (IQ) and strengths and difficulties questionnaire (SDQ) scores at preschool age. Independent variables included in model 4 gestational age, birth weight, small for gestational age (SGA), central venous catheter (CVC), sepsis, intraventricular hemorrhage (IVH), and age at full enteral feeding. For IQ and SDQ, parental educational levels were additional independent variables (model 5). Results are presented as β coefficients with 95% confidence intervals (CI) and corresponding p-values, with a forest plot depicting β and CI. Bold values indicate statistically significant differences (p < 0.05). *N* = 1975 for weight, *n* = 1989 for length, *n* = 1991 for head circumference, *n* = 1438 for IQ, *n* = 1489 for SDQ

## Discussion

CVCs are widely used in preterm infants. They are particularly valuable in the most immature neonates and those with highest morbidity, as they provide reliable vascular access and facilitate the administration of medications and highly dosed parenteral nutrition. However, in light of high utilization rates and substantial associated risks, including central line-associated BSI (CLABSI), the routine use of CVCs in the majority of preterm neonates warrants careful evaluation. As one of the first studies to assess long-term associations between CVC use and growth and neurodevelopment in a large cohort of preterm infants, our findings may contribute to a re-evaluation of the assumption that CVC use may confer general long-term developmental benefits in this population.

We have previously demonstrated that the delivery of parenteral nutrition is one of the main reasons for CVC insertion in preterm infants [7]. To ensure early provision of amino acids, lipids, and other essential nutrients in accordance with current guidelines [5, 6], the stepwise initial feeding advancement in preterm infants is commonly accompanied by parenteral nutrition, requiring peripheral or central venous access. Some studies have associated higher parenteral nutrient intakes with improved growth, but data are contradictory, and a benefit for long-term outcomes has not been confirmed to date [3, 4, 18–22]. Nonetheless, the delivery of highly concentrated parenteral nutrition, necessitating a CVC, has become standard practice in neonatal care. Furthermore, minimal handling and reduced interventions in VLBWI are considered protective and constitute additional important CVC indications [7, 23].

In a cohort of more than 2000 preterm children < 29 weeks of gestation, we assessed long-term growth as well as neurobehavioral outcome in infants with a history of CVC use and in those managed with peripheral venous access. Both groups were very heterogeneous, necessitating careful consideration of potential confounding factors, as infants receiving CVCs were generally more premature, had lower birth weights, and exhibited higher rates of prematurity-related morbidities. While unadjusted analyses suggested poorer long-term somatic and neurobehavioral outcomes in infants with CVC use, these associations were no longer observed after adjustment for gestational age and neonatal morbidity. in multivariable regression analyses accounting for CVC exposure and relevant confounders, outcomes were comparable between infants with and without CVC use, indicating no detectable association between CVC use and long-term somatic or neurodevelopmental outcomes. Propensity score matching confirmed these findings. Only BMI z-scores at preschool age were slightly higher in children without neonatal CVC use. The observed effect sizes were small, but may warrant further investigation regarding potential long-term metabolic effects of different nutritional strategies.

The current results add to our previous data, which indicated an association between CVC use and reduced short-term somatic growth velocity in VLBWI [Grimm and Retzmann et al., manuscript under review].

Given the potential contribution of early-life inflammatory insults to long-term outcomes [24], it remains plausible that complications potentially associated with CVC use could partially offset some of the intended benefits. Therefore, our findings suggest that a more restrictive approach to early-life CVC use may be justified. Instead of routine CVC placement, the use of peripheral venous catheters may be considered where clinically appropriate, and early advancement of enteral nutrition prioritized whenever feasible.

Notably, our regression analyses showed an inverse association between time to full enteral feeding and IQ at preschool age. While previous studies have suggested beneficial effects of early exposure to mother’s milk on neurodevelopment in preterm infants [25, 26], our results extend these findings by indicating, in a large cohort of VLBWI, that earlier attainment of full enteral feeding is associated with more favorable long-term cognitive outcome. These findings highlight early enteral feeding as a potential key modifiable factor in long-term development. In contrast, CVC use was not associated with corresponding long-term advantages in this study but, consistent with our previous work [Grimm and Retzmann et al., manuscript under review], was linked to delayed enteral feeding progression.

Previous studies have shown that early enteral nutrition and rapid feeding advancement can be safely implemented in preterm infants, with single trials using daily increments as high as 60 ml/kg and more [27–34]. Notably, the recent FEED1 trial including 2088 preterm infants born at 30–32 weeks of gestation demonstrated that initiating exclusive full milk feeds from the first day of life is safe compared with the traditional approach of intravenous fluids and gradual feeding advancement [35]. In contrast, slow feeding advancement delays full enteral nutrition and prolongs the use of intravenous catheters – with an increased risk of BSI associated with both peripheral and central venous access [29, 36]. BSI contribute significantly to neonatal mortality [37–39]. The reduction of catheter days is a crucial strategy to lower infection rates and can be supported by accelerating enteral feeding advancement [8, 40, 41].

Consistent with these data and current guidelines, our results support further reflection on widespread feeding strategies, suggesting that early advancement of enteral feeding in preterm infants may be preferable to prolonged reliance on parenteral nutrition whenever clinically feasible [22, 42].

This study is subject to several limitations. The selected follow-up cohort may introduce bias [43]. The study’s observational nature restricts analyses to associations, without permitting conclusions about causality. In addition, our data do not provide information on timing and indication for CVC insertion, nor can we confirm with certainty that all CVCs were used for parenteral nutrition. However, findings from our recent national survey indicate that most CVCs in VLBWI are placed within the first 3 days of life, which is within the main period of enteral feeding advancement [7]. We consider it therefore highly probable that the majority of indwelling catheters, even if inserted for other indications, were utilized for administering highly concentrated parenteral nutrition. We further acknowledge that important nutritional variables (e.g., the proportion of human milk versus formula feeding), were not available in the dataset and could therefore not be adjusted for, although these factors are known to influence long-term neurodevelopmental outcomes. Lastly, the heterogeneity between groups with and without CVC use represents a limitation of this study. Naturally, very immature infants or those with complications and severe morbidities are more likely to require a CVC during their hospital stay, but these conditions themselves might impact long-term outcome. To address this, we excluded infants with NEC or FIP and need for surgery, employed regression models to adjust for confounders, and performed propensity score matching to account for potential unknown variables. Across these statistical models, long-term growth and neurodevelopmental outcomes were comparable between children with and without CVC use. Nonetheless, residual confounding cannot be ruled out; in this case, comparable long-term outcomes in the more vulnerable CVC-exposed cohort may also be interpreted as a potential benefit of CVC use in this population. To further elucidate the impact of CVC use on nutrition and development of preterm infants, prospective trials are warranted that systematically compare feeding advancement, somatic growth, and long-term development.

Once again, these data underscore the increasingly recognized “less is more” principle in neonatal care. Emerging evidence indicates that minimizing interventions and invasive procedures is beneficial particularly for very immature preterm infants [40, 44]. In line with this concept, reducing routine early-life CVC insertion and prioritizing early advancement of enteral feeding may, where clinically feasible, improve long-term outcomes in preterm infants.

## Conclusion

While CVCs remain indispensable in selected critically ill or highly immature neonates, their use is associated with relevant risks. Given their widespread application in preterm infants, with indications including nutritional support, evaluation of potential long-term implications appears warranted. Within the limitations of this association study, our findings align with prior research, suggesting no clear long-term advantages in growth or neurodevelopment associated with CVC use. In contrast, earlier attainment of full enteral feeding was associated with more favorable neurodevelopmental outcome in this study.

Taken together, these findings support careful evaluation of routine CVC use in preterm infants by the means of device stewardship. Whenever clinically feasible, peripheral venous access and earlier enteral feeding advancement should be preferred, together with reduction of CVC exposure and duration, in order to reduce complications and improve long-term health in this vulnerable patient cohort.

## Supplementary information

Supplementary Material 1.

## Data Availability

The datasets analyzed during the current study are available from the authors upon reasonable request.

## Abbreviations

BMI: body mass index
BSI: blood stream infection
CI: confidence interval
CLABSI: central line-associated blood stream infection
CVC: central venous catheter
ESPGHAN: European Society for Paediatric Gastroenterology, Hepatology and Nutrition
FIP: focal intestinal perforation
GNN: German Neonatal Network
IQ: intelligence quotient
IVH: intraventricular hemorrhage
NEC: necrotizing enterocolitis
SD: standard deviation
SDQ: Strengths and Difficulties Questionnaire
SGA: small for gestational age
VLBWI: very low birth weight preterm infant
WPPSI III: Wechsler Preschool and Primary Scale – third edition

## References

[1] Humberg A, Fortmann I, Siller B, Kopp MV, Herting E, Göpel W et al (2020) Preterm birth and sustained inflammation: consequences for the neonate. Semin Immunopathol 42(4):451–468 32661735 10.1007/s00281-020-00803-2 PMC7508934

[2] Humberg A, Härtel C, Rausch TK, Stichtenoth G, Jung P, Wieg C et al (2020) Active perinatal care of preterm infants in the German Neonatal Network. Arch Dis Child Fetal Neonatal Ed 105(2):190–195 31248963 10.1136/archdischild-2018-316770

[3] Stephens BE, Walden RV, Gargus RA, Tucker R, McKinley L, Mance M et al (2009) First-Week Protein and Energy Intakes Are Associated With 18-Month Developmental Outcomes in Extremely Low Birth Weight Infants. Pediatrics 123(5):1337–1343 19403500 10.1542/peds.2008-0211

[4] Valentine CJ, Fernandez S, Rogers LK, Gulati P, Hayes J, Lore P et al (2009) Early amino-acid administration improves preterm infant weight. J Perinatol 29(6):428–432 19444236 10.1038/jp.2009.51 PMC2834366

[5] Lapillonne A, Fidler Mis N, Goulet O, van den Akker CHP, Wu J, Koletzko B (2018) ESPGHAN/ESPEN/ESPR/CSPEN guidelines on pediatric parenteral nutrition: Lipids. Clin Nutr 37(6 Pt B):2324–2336 30143306 10.1016/j.clnu.2018.06.946

[6] van Goudoever JB, Carnielli V, Darmaun D, Sainz de Pipaon M (2018) ESPGHAN/ESPEN/ESPR/CSPEN guidelines on pediatric parenteral nutrition: Amino acids. Clin Nutr 37(6 Pt B):2315–2323 30100107 10.1016/j.clnu.2018.06.945

[7] Retzmann J, Grimm A, Frieauff E, Schröder D, Dartsch S, Kampmeier S et al (2025) Central venous catheters in very low birthweight infants: results from a national survey. J Hosp Infect 165:73–80. 10.1016/j.jhin.2025.07.027. Epub 2025 Aug 11. PMID: 40803375 10.1016/j.jhin.2025.07.027 40803375

[8] Jansen SJ, Broer SDL, Hemels MAC, Visser DH, Antonius TAJ, Heijting IE et al (2024) Central-line-associated bloodstream infection burden among Dutch neonatal intensive care units. J Hosp Infect 144:20–27 38103692 10.1016/j.jhin.2023.11.020

[9] Hess S, Poryo M, Bottger R, Franz A, Klotz D, Linnemann K et al (2023) Umbilical venous catheter- and peripherally inserted central catheter-associated complications in preterm infants with birth weight < 1250 g: Results from a survey in Austria and Germany. Wien Med Wochenschr 173(7–8):161–167 35939216 10.1007/s10354-022-00952-z PMC10147741

[10] Goodman R (2001) Psychometric properties of the strengths and difficulties questionnaire. J Am Acad Child Adolesc Psychiatry 40(11):1337–1345 11699809 10.1097/00004583-200111000-00015

[11] Klasen H, Woerner W, Rothenberger A, Goodman R (2003) [German version of the Strength and Difficulties Questionnaire (SDQ-German)--overview and evaluation of initial validation and normative results]. Prax Kinderpsychol Kinderpsychiatr 52(7):491–502 14526759

[12] Geisler I, Rausch TK, Göpel W, Spiegler J (2021) Extremely and very preterm-born children < 1500 g show different weight development in childhood compared to their peers. Acta Paediatr 110(7):2093–2099 33533506 10.1111/apa.15785

[13] Cole TJ, Green PJ (1992) Smoothing reference centile curves: the LMS method and penalized likelihood. Stat Med 11(10):1305–1319 1518992 10.1002/sim.4780111005

[14] Voigt M, Rochow N, Schneider KT, Hagenah HP, Scholz R, Hesse V et al (2014) [New percentile values for the anthropometric dimensions of singleton neonates: analysis of perinatal survey data of 2007–2011 from all 16 states of Germany]. Z Geburtshilfe Neonatol 218(5):210–217 25353215 10.1055/s-0034-1385857

[15] Schwab F, Gastmeier P, Piening B, Geffers C (2012) The step from a voluntary to a mandatory national nosocomial infection surveillance system: the influence on infection rates and surveillance effect. Antimicrob Resist Infect Control 1(1):24 22958509 10.1186/2047-2994-1-24 PMC3489557

[16] Papile LA, Burstein J, Burstein R, Koffler H (1978) Incidence and evolution of subependymal and intraventricular hemorrhage: a study of infants with birth weights less than 1,500 gm. J Pediatr 92(4):529–534 305471 10.1016/s0022-3476(78)80282-0

[17] Austin PC (2011) An Introduction to Propensity Score Methods for Reducing the Effects of Confounding in Observational Studies. Multivar Behav Res 46(3):399–424 10.1080/00273171.2011.568786 PMC3144483 21818162

[18] Morgan C, McGowan P, Herwitker S, Hart AE, Turner MA (2014) Postnatal Head Growth in Preterm Infants: A Randomized Controlled Parenteral Nutrition Study. Pediatrics 133(1):e120–e8 24379229 10.1542/peds.2013-2207

[19] Smazal AL, Kavars AB, Carlson SJ, Colaizy TT, Dagle JM (2016) Peripherally inserted central catheters optimize nutrient intake in moderately preterm infants. Pediatr Res 80(2):185–189 27057735 10.1038/pr.2016.73

[20] Balakrishnan M, Jennings A, Przystac L, Phornphutkul C, Tucker R, Vohr B et al (2018) Growth and Neurodevelopmental Outcomes of Early, High-Dose Parenteral Amino Acid Intake in Very Low Birth Weight Infants: A Randomized Controlled Trial. JPEN J Parenter Enter Nutr 42(3):597–606 10.1177/0148607117696330 29187120

[21] Tesser F, Meneghelli M, Martino D, Pegoraro L, Pelosi MS, Sebellin S et al (2025) Early Optimal Parenteral Nutrition During NICU Stay and Neurodevelopmental Outcomes in Very Preterm Infants: State of the Art. Nutrients 17(2):232. PMID: 39861362; PMCID: PMC11767679 10.3390/nu17020232. PMID: 39861362; PMCID: PMC11767679.17(2) 10.3390/nu17020232 PMC11767679 39861362

[22] Robinson DT, Calkins KL, Chen Y, Cober MP, Falciglia GH, Church DD et al (2023) Guidelines for parenteral nutrition in preterm infants: The American Society for Parenteral and Enteral Nutrition. JPEN J Parenter Enter Nutr 47(7):830–858 10.1002/jpen.2550 37610837

[23] Schmid MB, Reister F, Mayer B, Hopfner RJ, Fuchs H, Hummler HD (2013) Prospective risk factor monitoring reduces intracranial hemorrhage rates in preterm infants. Dtsch Arztebl Int 110(29–30):489–496 24000297 10.3238/arztebl.2013.0489 PMC3752580

[24] Humberg A, Fortmann MI, Spiegler J, Rausch TK, Siller B, Silwedel C et al (2022) Recurrent Late-Onset Sepsis in Extremely Low Birth Weight Infants Is Associated with Motor Deficits in Early School Age. Neonatology 119(6):695–702 36327925 10.1159/000525709

[25] Bando N, Sato J, Vandewouw MM, Taylor MJ, Tomlinson C, Unger S et al (2024) Early nutritional influences on brain regions related to processing speed in children born preterm: A secondary analysis of a randomized trial. JPEN J Parenter Enter Nutr 48(7):778–786 10.1002/jpen.2669 39007723

[26] Bando N, Yoon EW, Beltempo M, de Cabo C, Colby L, Alburaki W et al (2025) Association of Enteral Feed Type with Neurodevelopmental and Neonatal Outcomes among Infants Born Preterm. J Pediatr 281:114536 40089177 10.1016/j.jpeds.2025.114536

[27] Morgan J, Young L, McGuire W (2015) Slow advancement of enteral feed volumes to prevent necrotising enterocolitis in very low birth weight infants. Cochrane Database Syst Reviews (10):CD001241. 10.1002/14651858.CD001241.pub6 10.1002/14651858.CD001241.pub6 26469124

[28] Dorling J, Hewer O, Hurd M, Bari V, Bosiak B, Bowler U et al (2020) Two speeds of increasing milk feeds for very preterm or very low-birthweight infants: the SIFT RCT. Health Technol Assess 24(18):1–94 32342857 10.3310/hta24180 PMC7212304

[29] Dorling J, Abbott J, Berrington J, Bosiak B, Bowler U, Boyle E et al (2019) Controlled Trial of Two Incremental Milk-Feeding Rates in Preterm Infants. N Engl J Med 381(15):1434–1443 31597020 10.1056/NEJMoa1816654

[30] Chitale R, Ferguson K, Talej M, Yang WC, He S, Edmond KM et al (2022) Early Enteral Feeding for Preterm or Low Birth Weight Infants: a Systematic Review and Meta-analysis. Pediatrics 150(Suppl 1):e2022057092E. 10.1542/peds.2022-057092E. PMID: 35921673 10.1542/peds.2022-057092E 35921673

[31] Söderquist Kruth S, Persad E, Rakow A (2025) Probiotic Supplements Effect on Feeding Tolerance, Growth and Neonatal Morbidity in Extremely Preterm Infants: A Systematic Review and Meta-Analysis. Nutrients 17(7):1228. 10.3390/nu17071228. PMID: 40218986; PMCID: PMC11990243 10.3390/nu17071228 PMC11990243 40218986

[32] Oddie SJ, Young L, McGuire W (2017) Slow advancement of enteral feed volumes to prevent necrotising enterocolitis in very low birth weight infants. Cochrane Database Syst Rev 8(8):Cd001241 28854319 10.1002/14651858.CD001241.pub7 PMC6483766

[33] Salas AA, Ojha S (2025) Exclusive enteral nutrition in preterm infants: How early is too early? Semin Fetal Neonatal Med 30(2):101631 40221313 10.1016/j.siny.2025.101631 PMC12162195

[34] Razzaghy J, Shukla VV, Gunawan E, Reeves A, Nguyen K, Salas AA (2024) Early and exclusive enteral nutrition in infants born very preterm. Archives Disease Child - Fetal Neonatal Ed 109(4):378–383 10.1136/archdischild-2023-325969 PMC11186726 38135494

[35] Ojha S, Mitchell EJ, Johnson MJ, Gale C, McGuire W, Oddie S et al (2025) Full exclusively enteral fluids from day 1 versus gradual feeding in preterm infants (FEED1): a open-label, parallel-group, multicentre, randomised, superiority trial. Lancet Child Adolesc Health 9(12):827–836 41115446 10.1016/S2352-4642(25)00271-8

[36] Geffers C, Gastmeier A, Schwab F, Groneberg K, Rüden H, Gastmeier P (2010) Use of central venous catheter and peripheral venous catheter as risk factors for nosocomial bloodstream infection in very-low-birth-weight infants. Infect Control Hosp Epidemiol 31(4):395–401 20175683 10.1086/651303

[37] Stichtenoth G, Demmert M, Bohnhorst B, Stein A, Ehlers S, Heitmann F et al (2012) Major contributors to hospital mortality in very-low-birth-weight infants: data of the birth year 2010 cohort of the German Neonatal Network. Klin Padiatr 224(4):276–281 22441803 10.1055/s-0032-1306344

[38] Köstlin-Gille N, Härtel C, Haug C, Göpel W, Zemlin M, Müller A et al (2021) Epidemiology of Early and Late Onset Neonatal Sepsis in Very Low Birthweight Infants: Data From the German Neonatal Network. Pediatr Infect Dis J 40(3):255–259 33538544 10.1097/INF.0000000000002976

[39] Härtel C, Faust K, Fortmann I, Humberg A, Pagel J, Haug C et al (2020) Sepsis related mortality of extremely low gestational age newborns after the introduction of colonization screening for multi-drug resistant organisms. Antimicrob Resist Infect Control 9(1):144 32843080 10.1186/s13756-020-00804-8 PMC7449086

[40] Shin J, Kang HM, Kim SY, Youn YA, Choi CW, Chang YS (2024) The effect of minimizing central line days for very low birth weight infants through quality improvement. Sci Rep 14(1):3854 38360733 10.1038/s41598-024-53163-4 PMC10869738

[41] Milstone AM, Reich NG, Advani S, Yuan G, Bryant K, Coffin SE et al (2013) Catheter dwell time and CLABSIs in neonates with PICCs: a multicenter cohort study. Pediatrics 132(6):e1609–e1615 24218474 10.1542/peds.2013-1645 PMC3838533

[42] Embleton ND, Jennifer Moltu S, Lapillonne A, van den Akker CHP, Carnielli V, Fusch C et al (2023) Enteral Nutrition in Preterm Infants (2022): A Position Paper From the ESPGHAN Committee on Nutrition and Invited Experts. J Pediatr Gastroenterol Nutr 76(2):248–268 36705703 10.1097/MPG.0000000000003642

[43] Humberg A, Spiegler J, Fortmann MI, Zemlin M, Marissen J, Swoboda I et al (2020) Surgical necrotizing enterocolitis but not spontaneous intestinal perforation is associated with adverse neurological outcome at school age. Sci Rep 10(1):2373 32047169 10.1038/s41598-020-58761-6 PMC7012917

[44] Härtel C, Paul P, Hanke K, Humberg A, Kribs A, Mehler K et al (2018) Less invasive surfactant administration and complications of preterm birth. Sci Rep 8(1):8333 29844331 10.1038/s41598-018-26437-x PMC5974027

