# Supplementary material for: The impact of central venous catheter use on long-term growth and neurobehavioral outcome in preterm children born < 29 weeks of gestation
Source: Mol Cell Pediatr. 2026 Jun 23;13:35. doi: 10.1186/s40348-026-00247-y (PMC13291290; doi:10.1186/s40348-026-00247-y)
Supplement: Supplementary file 1 — Supplementary Material 1. [file 40348_2026_247_MOESM1_ESM.docx]

**Additional Files**

**The impact of central venous catheter use on growth and neurobehavioral outcome in preterm children born < 29 weeks of gestation**

Christine Silwedel, Jana Retzmann, Ingmar Fortmann, Anne Grimm, Anna Häfke, Fabian Kleindiek, Kathrin Hanke, Cornelia Wiechers, Ursula Felderhoff-Müser, Harald Ehrhardt, Jochen Essers, Egbert Herting, Juliane Spiegler, Wolfgang Göpel, Christoph Härtel

| **Additional table 1:** Multiple linear regression analysis assessing long-term growth (model 1) | | | | | | | |
| --- | --- | --- | --- | --- | --- | --- | --- |
|  | | **Dependent variable (follow-up)** | | | | | |
|  |  | **Weight (kg)** | | **Length (cm)** | | **Head circumference (cm)** | |
|  |  | **β**  **(95 % CI)** | **p** | **β**  **(95 % CI)** | **p** | **β**  **(95 % CI)** | **p** |
| Independent variable | Gestational age (weeks) | -0.457  (-0.595- -0.319) | **<0.001** | -0.726  (-0.965- -0.487) | **<0.001** | -0.137  (-0.208- -0.067) | **<0.001** |
|  | Birth weight (g) | 0.006  (0.005-0.007) | **<0.001** | 0.010  (0.008-0.012) | **<0.001** | 0.004  (0.003-0.004) | **<0.001** |
|  | SGA | -0.545  (-1.094-0.004) | 0.052 | -1.142  (-2.096- -0.188) | **0.019** | -0.554  (-0.835- -0.273) | **<0.001** |
|  | CVC | 0.018  (-0.331-0.368) | 0.917 | 0.554  (-0.052-1.159) | 0.073 | -0.090  (-0.268-0.088) | 0.322 |
|  | Sepsis | -0.160  (-0.558-0.237) | 0.430 | -0.251  (-0.944-0.442) | 0.478 | -0.182  (-0.386-0.022) | 0.081 |
|  | IVH | -0.548  (-0.903- -0.194) | **0.002** | -0.806  (-1.420- -0.192) | **0.010** | -0.406  (-0.587- -0.226) | **<0.001** |

Linear regression analysis was conducted to evaluate weight, body length, and head circumference at preschool age, respectively, as dependent variables. Independent variables included gestational age, birth weight, small for gestational age (SGA), central venous catheter (CVC), sepsis, and intraventricular hemorrhage (IVH). Results are presented as β coefficients with 95% confidence intervals (CI) and corresponding p-values, with β coefficients > 0 indicating higher growth parameters. N = 2,049 for weight, 2,062 for length, 2,065 for head circumference.

| **Additional table 2:** Multiple linear regression analysis assessing BMI at follow-up (model 1) | | | | | |
| --- | --- | --- | --- | --- | --- |
|  | | **Dependent variable (follow-up)** | | | |
|  |  | **BMI (kg/m^2^)** | | **Z-score BMI** | |
|  |  | **Β (95 % CI)** | **p** | **Β (95 % CI)** | **p** |
| Independent variable | Gestational age (weeks) | -0.148 (-0.216- -0.080) | **<0.001** | -0.113 (-0.157- -0.069) | **<0.001** |
|  | Birth weight (g) | 0.002 (0.002-0.003) | **<0.001** | 0.001 (0.001-0.002 | **<0.001** |
|  | SGA | -0.207 (-0.476-0.062) | 0.131 | -0.184 (-0.357- -0.010) | **0.038** |
|  | CVC | -0.122 (-0.293-0.049) | 0.163 | -0.123 (-0.233- -0.012) | **0.030** |
|  | Sepsis | -0.076 (-0.271-0.119) | 0.447 | -0.081 (-0.207-0.045) | 0.209 |
|  | IVH | -0.058 (-0.129-0.013) | 0.111 | -0.056 (-0.102- -0.010) | **0.017** |

Linear regression analysis was conducted to evaluate BMI and BMI z-scores at preschool age as the dependent variables, both adjusted for gestational age. Independent variables included gestational age, birth weight, small for gestational age (SGA), central venous catheter (CVC), sepsis, and intraventricular hemorrhage (IVH). Results are presented as β coefficients with 95% confidence intervals (CI) and corresponding p-values. N = 2,041 for BMI, n = 1,996 for z-score BMI.

| **Additional table 3:** Multiple linear regression analysis evaluating long-term neurological outcome (model 2) | | | | | | | |
| --- | --- | --- | --- | --- | --- | --- | --- |
|  | | **Dependent variable (follow-up)** | | | |  |  |
|  |  | **IQ** | | **SDQ** | |  |  |
|  |  | **β**  **(95 % CI)** | **p** | **β**  **(95 % CI)** | **p** |  |  |
| Independent variable | Gestational age (weeks) | 1.175 (0.569-1.780) | **<0.001** | -0.621 (-0.877- -0.365) | **<0.001** |  |  |
|  | Birth weight (g) | 0.004 (-0.001-0.008) | 0.088 | 0.000 (-0.002-0.002) | 0.905 |  |  |
|  | SGA | -3.145 (-5.610- -0.680) | **0.012** | 0.956 (-0.076-1.988) | 0.069 |  |  |
|  | CVC | -0.136 (-1.607-1.334) | 0.856 | -0.068 (-0.709-0.573) | 0.836 |  |  |
|  | Sepsis | -0.551 (-2.303-1.201) | 0.537 | 0.830 (0.096-1.565) | **0.027** |  |  |
|  | IVH | -3.413 (-4.996- -1.831) | **<0.001** | 1.100 (0.449-1.751) | **<0.001** |  |  |
|  | Educational level mother | 0.079 (0.053-0.105) | **<0.001** | -0.018 (-0.029- -0.007) | **0.001** |  |  |
|  | Educational level father | 0.060 (0.034-0.086) | **<0.001** | -0.019 (-0.030- -0.008) | **<0.001** |  |  |

Linear regression analysis was conducted to evaluate intelligence quotient (IQ) and strengths and difficulties questionnaire (SDQ) at preschool age as dependent variables. Independent variables included gestational age, birth weight, small for gestational age (SGA), central venous catheter (CVC), sepsis, intraventricular hemorrhage (IVH), maternal and paternal educational levels. Results are presented as β coefficients with 95% confidence intervals (CI) and corresponding p-values, with β coefficients > 0 indicating higher scores. N = 1,489 for IQ and n = 1,539 for SDQ.

| **Additional table 4:** Propensity score matching (model 3) | | | | | | | | | | | | | | | |
| --- | --- | --- | --- | --- | --- | --- | --- | --- | --- | --- | --- | --- | --- | --- | --- |
|  | **Baseline characteristics at follow-up (number of infant pairs)** | | | | | | | | | | | | | |  |
|  | **Weight**  **(483)** | | **Length**  **(488)** | | **Head cir-cumference (486)** | | **BMI**  **(483)** | | **Z-score BMI (473)** | | **IQ**  **(430)** | | **SDQ**  **(412)** | |  |
|  | SMD pre | SMD post | SMD pre | SMD post | SMD pre | SMD post | SMD pre | SMD post | SMD pre | SMD post | SMD pre | SMD post | SMD pre | SMD post |  |
| Gender (male/female) | 0.09 | 0.00 | 0.09 | 0.01 | 0.09 | 0.00 | 0.09 | 0.00 | 0.07 | 0.00 | 0.06 | 0.01 | 0.07 | 0.00 |  |
| Gestational age | 0.90 | 0.01 | 0.89 | 0.00 | 0.89 | 0.00 | 0.90 | 0.01 | 0.90 | 0.01 | 0.84 | 0.03 | 0.89 | 0.01 |  |
| SGA (yes/no) | 0.34 | 0.01 | 0.33 | 0.01 | 0.33 | 0.02 | 0.34 | 0.01 | 0.35 | 0.01 | 0.37 | 0.02 | 0.31 | 0.01 |  |
| Maternal education |  |  |  |  |  |  |  |  |  |  |  |  |  |  |  |
| High | 0.04 | -0.02 | 0.05 | 0.00 | 0.05 | 0.00 | 0.05 | 0.00 | 0.04 | -0.02 | 0.08 | 0.01 | 0.05 | -0.01 |  |
| Medium | 0.02 | 0.00 | 0.01 | -0.01 | 0.01 | -0.02 | 0.01 | -0.01 | 0.03 | 0.00 | 0.00 | 0.00 | 0.04 | 0.01 |  |
| Low | -0.08 | 0.02 | 0.08 | 0.02 | 0.08 | 0.02 | -0.08 | 0.02 | 0.10 | 0.02 | -0.10 | -0.01 | -0.14 | 0.02 |  |
| IVH (yes/no) | 0.35 | 0.03 | 0.34 | 0.01 | 0.34 | 0.01 | 0.34 | 0.03 | 0.34 | 0.02 | 0.31 | 0.04 | 0.41 | 0.01 |  |

Propensity score matching was conducted using gender, gestational age, small for gestational age (SGA), maternal educational level (high: upper secondary education, “Abitur”; medium: lower secondary education, “Realschulabschluss”; low: other or no completed school education), and intraventricular hemorrhage (IVH). Baseline characteristics assessed at follow-up included weight, length, head circumference, body mass index (BMI) and its gestational age-adjusted z-scores, intelligence quotient (IQ), as well as strengths and difficulties questionnaire (SDQ) scores. Standardized mean differences (SMD) are reported pre and post matching to confirm full matching. For binary variables (gender, SGA, IVH), absolute SMD values are given.

| **Additional table 5:** Multiple linear regression analysis assessing initial enteral feeding advancement (model 1). | | | |
| --- | --- | --- | --- |
|  | | **Dependent variable: full enteral feeding (d)** | |
|  |  | **Β (95 % CI)** | **p** |
| Independent variable | Gestational age (weeks) | -0.117 (-1.769- -0.561) | **<0.001** |
|  | Birth weight (g) | -0.033 (-0.006-0.002) | 0.367 |
|  | SGA | 0.038 (-0.710-4.105) | 0.167 |
|  | CVC | 0.187 (4.864-7.917) | **<0.001** |
|  | Sepsis | 0.060 (0.687-4.179) | **0.006** |
|  | IVH | 0.030 (-0.476-2.623) | 0.174 |

Multiple linear regression analysis was conducted to evaluate full enteral feeding as the dependent variable. Independent variables included gestational age, birth weight, small for gestational age (SGA), central venous catheter (CVC), sepsis, and intraventricular hemorrhage (IVH). Results are presented as β coefficients with 95% confidence intervals (CI) and corresponding p-values, with β coefficients > 0 indicating a higher number of days. N = 2,072.
